# Effect of one prophylactic dose of azithromycin on *Bifidobacterium infantis* colonization in infants from the Mumta trial

**DOI:** 10.1016/j.ijid.2025.107794

**Published:** 2025-04

**Authors:** Aneela Pasha, Najeeha Talat Iqbal, Yasir Shafiq, Waqasuddin Khan, Syed Iqbal Azam, Furqan Kabir, Ameer Muhammad, Muhammad Imran Nisar, Fyezah Jehan

**Affiliations:** 1 Department of Paediatrics and Child Health, The Aga Khan University, Karachi, Pakistan; 2 Department of Biological & Biomedical Sciences, The Aga Khan University, Karachi, Pakistan; 3 Center of Excellence for Trauma and Emergencies and Community Health Sciences, The Aga Khan University, Karachi, Pakistan; 4 Harvard T. H. Chan School of Public Health, Boston, Massachusetts, USA; 5 Department of Community Health Sciences, The Aga Khan University, Karachi, Pakistan; 6 Vital Pakistan Trust, Karachi, Pakistan

**Keywords:** Azithromycin, *Bifidobacterium infantis*, Microbiota, Infant, Antimicrobial resistance

## Abstract

**Objectives:**

The effects of antibiotics on the microbiome remain incompletely understood. Azithromycin (AZ) has been shown to improve child survival and infant growth outcomes. This study aimed to assess the impact of AZ on *Bifidobacterium infantis* colonization and bacterial enteropathogen count in the infant gut.

**Methods:**

We analyzed clinical, biomarker, *B. infantis* and enteropathogen data from 150 mother-infant dyads from the MUMTA Lactating Women study. Colonization of *B. infantis* was assessed using quantitative polymerase chain reaction (PCR) of fecal samples. We utilized a customized PCR-based TaqMan Array Card for enteropathogen detection.

**Results:**

AZ administration was associated with a 1.99-fold (95% confidence intervals [CI] 1.33-2.97) increase in colonization by *B. infantis. B. infantis* colonization was highest when inflammatory biomarker levels were within normal range. Mode of delivery (RR 2.43; 95% CI: 1.58, 3.76) and colostrum (RR2.05; 95% CI: 1.41, 2.98) given to the infant within 24 h of birth were associated with *B. infantis* colonization. A single dose of AZ on day 42 reduced bacterial enteropathogen count in the AZ group on day 56, as compared to the pre-AZ count. Bacterial enteropathogen count for infants with wasting (weight for length *z*-score WLZ <−2) was 1.43-fold higher (95% CI: 1.00-2.03) than for infants with WLZ ≥ −2. Over 60% of infants harbored with the macrolide resistance mph(A) gene

**Conclusion:**

AZ administration increases *B. infantis* colonization and reduces bacterial enteropathogen count in infants.

## Highlights

• Prophylactic azithromycin to infants enhances colonization by *B. infantis*.
• *B. infantis* thrives in infant's gut when inflammatory levels are in normal range.
• Infants with wasting (WLZ <−2) have higher bacterial count than those with WLZ ≥−2.
• Bacterial counts decrease in infants after prophylactic dose of azithromycin.
• Large number of healthy infants harbor macrolide resistance mph(A) gene.

## Graphical abstract

**Figure:**
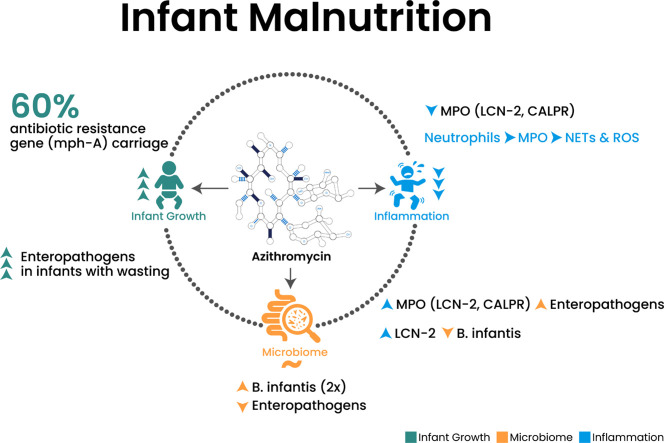

## Introduction

Azithromycin (AZ) is an effective macrolide antibiotic in the World Health Organization (WHO) framework for improving child survival. Recently, the WHO issued guidelines [1] on the mass drug administration (MDA) of AZ for distribution to communities with high infant mortality. AZ has been used in children in Sub Saharan Africa for treatment of trachoma and yaws [2], and in newborns to reduce infection related morbidity and mortality [3].

*Bifidobacterium Longum* subspecies *infantis* (*B. infantis*) colonization in the infant gut is a primary signature of a healthy microbiome. *B. infantis* is highly prevalent in the infant gut, particularly in breastfed infants. In countries where breastfeeding rates are high (such as Bangladesh), the prevalence of *B. infantis* colonization is over 60% [4]. Conversely, in countries where breastfeeding rates are low (such as the USA), the prevalence of *B. infantis* colonization can be as low as 0% [4]. The major contribution of *B. infantis* in the infant gut is the metabolic breakdown of human milk oligosaccharides (HMOs) and conditioning of the infant's immune system [5].

Disturbances in microbiota composition and diversity (i.e., dysbiosis) have been implicated in several diseases, as well as malnutrition, Environmental Enteric Dysfunction (EED), and stunted growth. Few studies have assessed the impact of AZ on gut microbiota in infants. A recent review identified five randomized controlled trials from Niger, Burkina Faso, and India that examined the short-term effects of prophylactic MDA-AZ on gut microbiota [6]. These studies found that AZ was associated with a decrease in the α-diversity index and a reduction in pathogens from the *Campylobacter* and *Enterobacteriaceae* families.

In this sub-study of infants from the Mumta trial [7], we assessed the short-term impact of one prophylactic dose of AZ given to infants at 42 days postbirth on *B. infantis* colonization and bacterial count 2 weeks later (56 days postbirth). We also assessed the influence of other factors (such as mode of delivery and biomarkers of anemia and inflammation) on the colonization of *B. infantis*. Results of this analysis could provide clues to the mechanism of action of AZ, along with the health benefits observed after AZ administration in malnourished infants.

## Methodology

### Study design and intervention

The Mumta trial has been described in detail previously [7]. Briefly, Mumta was a randomized controlled trial conducted between 2018 and 2020 in 3 peri‑urban communities of Karachi. It was a 3-arm, open-label, assessor-blinded trial with a treatment allocation ratio of 1:1:1 (*n* = 957).

Mothers randomized to the first (control) arm received counseling on lactation, nutrition, infant immunization, and health promotion plus iron-folate supplementation until the infant was 6 months of age. Mothers randomized to the second arm received counseling, as well as two 75-g sachets of BEP per day starting from the time of enrollment until the infant was 6 months of age. In the third arm, mothers received the same counseling and BEP sachets, and the infant received 1 dose of prophylactic oral AZ (at 20 mg/km) at 42 days of life, with a window period of ±7 days (BEP plus AZ arm).

From this sample, a random subsample of 50 mothers per arm enrolled in the sub-study. The random selection of mothers was carried out using block randomization with a block size of 6.

### Study outcomes and measurement

The blood biomarkers C-reactive protein (CRP), alpha-1 acid glycoprotein (AGP), hemoglobin, ferritin (FER), and soluble transferrin receptor (sTfR) were measured. Stool samples were assessed for biomarkers myeloperoxidase (MPO), fecal calprotectin (CALPR) and lipocalin-2 (LCN-2), presence of enteropathogens and presence of *Bifidobacteria* subspecies *infantis* and *longum*.

The primary objective of the study was to determine the association between AZ and *B. infantis* colonization. The main outcome was the colonization of *B. infantis* in the infant's stool (yes/no), measured at 56 days since birth (post-AZ) by quantitative polymerase chain reaction. *Bifidobacterium* 16S rDNA copy numbers (per gram of stool) were measured with a cycle threshold (CT) of 34 (CT <34 was taken to indicate that *Bifidobacterium* was present). Several covariates were considered: place and mode of delivery, baseline mother and infant anthropometric measures, colostrum (given to infant within 24 h of birth, antibiotics given to infant (not as part of the trial), and biomarkers measured at the 56-day timepoint.

The secondary objective was to compare bacterial count in infants by treatment arm. The main outcome variable was the bacterial count post-AZ. Enteropathogen detection was done using a customized TaqMan Array Card (TAC), previously described in detail [8]. The enteric pathogen panel includes viruses, bacteria, helminths, and antimicrobial resistance genes (ARGs). A CT of 34 was applied to indicate the presence (CT < 34) of enteropathogens. The same covariates were considered in this analysis. ARGs were also compared by treatment arm.

### Statistical analysis

All analyses were done using STATA version 17.0, developed by Stata Corp LLC, located in College Station, Texas, USA [9]. For all categorical participant characteristics (such as infant gender and mode of delivery), frequencies and percentages were computed. For quantitative variables (such as infant weight at baseline), mean and standard deviations were computed. Median and interquartile ranges were calculated for biomarkers. Due to the skewed distributions of most biomarkers, four quartiles were generated for each biomarker (q1: 0-25th percentile; q2: 25th-50th quartile; q3: 50th-75th quartile; q4: 75th-100th percentile). The biomarkers were considered categorical predictors, and the lowest quartile (q1) was used as the reference group. WHO criteria for infant growth were used to calculate *z*-scores, which were assessed as both continuous and categorical predictors. Underweight was categorized as weight-for-age *z*-score (WAZ) <−2 SD. Stunting was categorized as length for age *z*-score <−2 SD. Wasting was categorized as weight for length *z*-score (WLZ) <−2 SD. Maternal body mass index was categorized using WHO criteria as underweight <18.5 and normal weight ≥18.5. Mid-upper arm circumference was categorized as <21 cm and ≥21 cm. Maternal age was categorized as a dichotomous predictor (<30 years and ≥30 years).

The chi-square statistical test was used to infer differences across categorical covariates (such as mode of delivery) in the three arms. One-way ANOVA was used to infer differences across continuous covariates (such as length of infant) and the Kruskal-Wallis test was used to assess differences between biomarkers in the three arms. Spearman correlations were estimated for infant and maternal biomarkers at day 56 (post-AZ).

The Cox proportional algorithm was used to estimate the association between a single dose of AZ and colonization of *B. infantis* (yes/no). We utilized a backward stepwise regression approach to identify the most significant predictors. Covariates were iteratively removed based on their *P*-values, starting with the least significant covariate. A significance level of 0.05 was used as the threshold for retaining covariates in the model. After each elimination step, the model was re-evaluated to ensure that the proportional hazards assumption was not violated and that the model adequately fit the data. Due to the presence of extreme values in biomarker data, as well as anthropometric measurements, adjustment through robust regression was applied. Interpretations were based on statistical significance as well as biological plausibility. The final model significance was checked through the model likelihood ratio and *P*-value (<0.05).

For the secondary objective, 26 bacterial targets from TAC were included (Table S1). The total number of bacterial targets in each infant sample was counted to create a linear variable. A generalized linear model (negative binomial regression) was then used to model the bacterial count as the dependent variable.

Similar to the Cox Proportional Algorithm model building strategies, a backward stepwise regression approach was used to identify the most significant predictors. Incidence rate ratios and 95% confidence intervals (CI) were calculated for each covariate in the final model.

## Results

**Figure 1:**
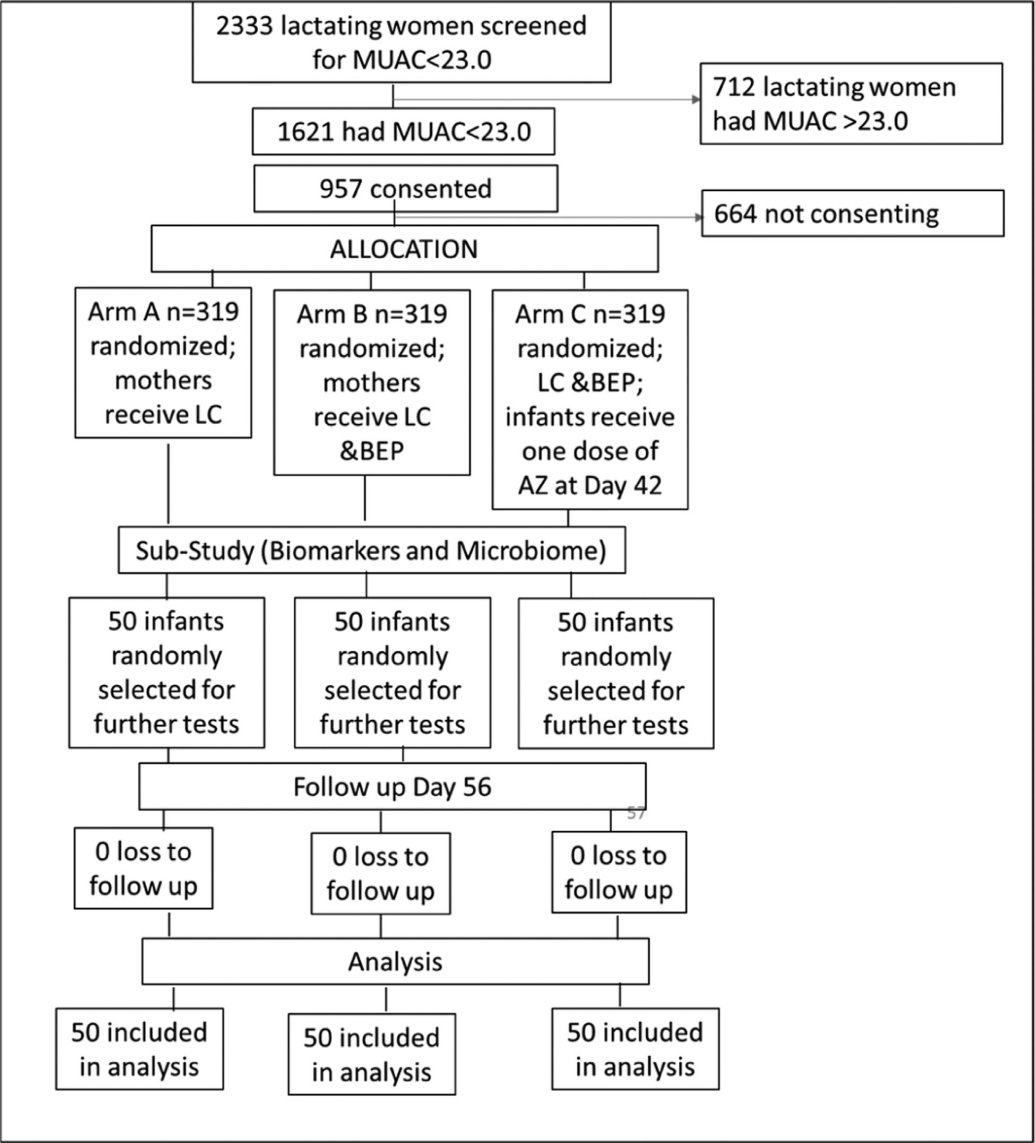
Flowchart of participants in MUMTA Lactating Woman sub-study.

A total of 50 infants from each arm were included in this analysis (Figure 1). The infants in the three trial arms were similar in baseline anthropometric measures, maternal and clinical characteristics (such as mode of delivery and feeding patterns) (Table 1). In the present analysis, 84 (56%) of the infants were female. The average gestational age at birth was 39.1 ± 1.5 weeks, and the proportion of preterm births was 33 out of 150.

**Table 1:** Baseline anthropometric measurements and feeding practices by treatment arm.

Variable	Total *N* = 150	Control arm (*n* = 50)	BEP alone arm (*n* = 50)	BEP plus AZ arm (*n* = 50)
Female *n* (%)	84 (56)	29 (58)	24 (48)	31 (62)
Male *n* (%)	66 (44)	21 (42)	26 (52)	19 (38)
Gestational age (weeks) (mean ± SD)	39.1 ± 1.5	39.3 ± 1.5	38.9 ± 1.6	38.9 ± 1.5
Preterm ≤37 weeks gestation *n* (%)	33 (22)	7 (14)	13 (30)	11 (22)
**Baseline infant anthropometry (mean ± SD)**				
Weight (g)	2792.1 ± 409.4	2749.7 ± 434.9	2853.5 ± 387.4	2773.2 ± 405.6
Length (cm)	48.3 ± 1.8	48.2 ± 1.9	48.6 ± 1.7	48.2 ± 1.9
MUAC (cm)	9.4 ± 0.8	9.3 ± 0.7	9.5 ± 0.8	9.5 ± 0.7
OF head circumference (cm)	33.1 ± 1.2	32.8 ± 1.1	33.3 ± 1.3	33.2 ± 1.3
**Infant clinical characteristics *n* (%)**				
Stunting (LAZ <−2)	22 (14.7)	10 (20)	3 (6)	9 (18)
Wasting (WLZ <−2)	27 (18)	11 (22)	8 (16)	8 (16)
Underweight (WAZ <−2)	32 (21.3)	13 (26)	7 (14)	12 (24)
**Maternal characteristics**				
Age <30 years *n* (%)	124 (83)	40 (80)	37 (74)	47 (94)
Age >30 years *n* (%)	26 (17)	10 (20)	13 (26)	3 (6)
Height (cm) mean ± SD	152.4 ± 5.8	152.8 ± 5.3	151.4 ± 5.7	152.8 ± 6.3
BMI mean ± SD	19.8 ± 1.7	19.4 ± 1.4	20.2 ± 1.7	19.7 ± 1.9
Place of delivery *n* (%)				
Hospital/clinic	111 (74)	13 (26)	12 (24)	14 (28)
Home	39 (26)	37 (74)	38 (76)	36 (72)
Mode of delivery *n* (%)				
Vaginal	126 (63)	46 (92)	40 (80)	40 (80)
C-section	24 (37)	4 (8)	10 (20)	10 (20)
Total # of pregnancies mean ± SD	3.1 ± 2.1	3.0 ± 2.0	3.6 ± 2.8	2.6 ± 1.4
**Feeding practices**				
Colostrum postdelivery *n* (%)				
<2 h of birth	82 (55)	8 (16)	13 (26)	9 (18)
>2 h of birth	68 (45)	42 (84)	37 (74)	41 (82)
Prelacteal feed *n* (%) yes	93 (62)	32 (64)	29 (58)	32 (64)
No	57 (38)	18 (36)	21 (42)	18 (16)
EBF compliance *n* (%) yes	135 (90)	42 (84)	45 (90)	48 (96)
No	15 (10)	8 (16)	5 (10)	2 (4)
Drinking boiled water *n* (%) yes	30 (20)	8 (16)	13 (26)	9 (18)
No/sometimes	120 (80)	42 (84)	37 (74)	41 (82)
***Bifidobacterium infantis* colonization *n* (%)**				
Yes	122 (81.3)	41 (82)	35 (70)	46 (92)
No	28 (18.7)	9 (18)	15 (30)	4 (8)

BMI, body mass index; EBF, exclusive breastfeeding; LAZ, length for age *z*-score; MUAC, mid-upper arm circumference; OF, occipital-frontal; WAZ, weight for age *z*-score; WLZ, weight for length *z*-score.

A total of 22 of 150 infants (14.7%) had moderate-to-severe stunting (9 infants of the 22 were in the AZ arm), 27 of 150 (18%) had moderate-to-severe wasting (8 infants of the 27 were in the AZ arm).

Among feeding practices, breastfeeding postdelivery was evaluated by colostrum given to the infant within 2 hours of birth (“immediate”) or more than 2 hours after birth (“delayed”). The median time of breastfeeding was 2 h postbirth, with greater than 90% of mothers breastfeeding the newborn in all three groups. Interestingly, 62% reported giving the infant prelacteal feeds, the most common being honey (40%), tea (27%), formula milk (17%), Ghutti (13%), water (6%), and glucose water (6%).

The prevalence of *B. infantis* (primary outcome) in the 150 infants was 81.3%. The prevalence of *B. infantis* in the AZ arm was 92%, significantly higher than in the non-AZ arms (*P* = 0.02).

### Descriptive analysis of mother and infant biomarker data

Median values with a range of biomarker levels after AZ administration are presented in Table 2 by treatment arm and overall numbers. In this sub-sample, maternal sTfR was significantly lower in Arm 3 (*P* = 0.005). Post-AZ, median CALPR and MPO levels in infants were lower in the AZ treatment arm as compared to the other groups (*P* = 0.005 and *P* = 0.06, respectively).

**Table 2:** Descriptive analysis of stool and systemic biomarkers taken at day 56 postbirth (post-AZ) of mother and infant by treatment arm.

Biomarker median (range)	Total *N* = 150	Control arm (*n* = 50)	BEP alone arm (*n* = 50)	BEP plus AZ arm (*n* = 50)	*P-*value
**Maternal stool**					
CALPR (µg/gm)	4.0 (1.5-10.8)	5.8 (1.7-13.5)	3.5 (1.2-7.9)	3.6 (1.6-11.0)	0.3
LCN2 (ng/gm)	286.7 (90.0-834.2)	451.7 (151.3-1692.4)	203.2 (58.8-589.9)	233.9 (87.7-473.1)	0.1
MPO (ng/mL)	416.3 (86.7-2490.5)	540.1 (93.9-2577.5)	242.1 (75.0-2220.5)	412.0 (86.4-2155.5)	0.6
**Maternal systemic**					
Hgb (gm/dL)	12.3 (11.5-12.9)	12.2 (11.2-12.8)	12.3 (11.7-12.8)	12.4 (11.4-13.1)	0.6
FER (ng/mL)	52.9 (23.9-115.9)	50.2 (19.7-86.1)	52 (31.0-119.5)	62.8 (30.1-181.3)	0.1
sTfR (mg/L)^a^	3.8 (2.6-5.4)	4.3 (3.1-6.5)	3.2 (2.3-4.9)	3.4 (2.4-4.3)	0.005^a^
CRP (mg/dL)	0.1 (0.06-0.29)	0.1 (0.06-0.31)	0.1 (0.06-0.24)	0.1 (0.04-0.33)	0.8
AGP (mg/dL)	86.5 (74-108)	86 (74-108)	90.5 (72-108)	86.5 (76-111)	0.9
**Infant stool**					
CALPR (µg/gm)^a^	38.7 (16.7-87.7)	56.1 (31.5-126.3)	34.6 (7.5-81.5)	24.6 (12-56.4)	0.005^a^
LCN2 (ng/gm)	11,696.9 (7828.3-17,731.4)	11,502.9 (7793.3-16,040.2)	11,879.3 (7974-20,860.8)	11,802.7 (7717.4-17,510.1)	0.5
MPO (ng/mL)	4620.8 (785.2-13,817.5)	4419 (1208.5-1397.6)	6237.7 (2260.5-1707.1)	2234.2 (764.8-5855)	0.06
**Infant systemic**					
Hgb (gm/dL)	10.9 (10.2-11.8)	10.9 (10.2-11.8)	10.7 (10.1-11.6)	10.8 (10.3-11.9)	0.7
FER (ng/mL)	295.2 (191.9-394)	342.5 (200.2-532.3)	291.1 (191.8-375.7)	274.5 (194.1-331.7)	0.1
sTfR (mg/L)	3.1 (2.6-3.9)	3.1 (2.6-3.8)	3.1 (2.6-3.6)	3.1 (2.8-4)	0.4
CRP (mg/dL)	0.07 (0.03-0.16)	0.08 (0.04-0.15)	0.06 (0.03-0.2)	0.07 (0.03-0.3)	0.7
AGP (mg/dL)	63 (44-82)	70.5 (48-94)	54 (41-78)	60.5 (45-82)	0.09

AGP, alpha glycoprotein-1; CALPR, fecal calprotectin; CRP, C-reactive protein; FER, ferritin; Hgb, hemoglobin; LCN2, lipocalin-2; MPO, myeloperoxidase; sTfR, soluble transferrin receptor.

a *P-*value <0.05. Kruskal Wallis test was used to compare medians of biomarker levels.

### Impact of AZ on *B. infantis* colonization in infants at day 56 postbirth

Adjusted RR with 95% CI for variables predicting *B. infantis* colonization of the infant's gut after AZ are shown in Table 3. The most important infant predictors were exposure to AZ, infant MPO, and LCN-2 levels. In infants who were given a single dose of AZ, *B. infantis* colonization was 1.99 times higher as compared to those who were not (95% CI 1.33-2.97). In infants with MPO in the second quartiles (785.2-4620.75 ng/mL) *B. infantis* colonization was 2.84 times (95%CI 1.72-4.69) higher as compared to the reference (first quartile). Elevated LCN-2 levels were associated with 49% reduction in *B. infantis* colonization (95% CI 0.31-0.81).

**Table 3:** Factors associated with *Bifidobacterium infantis* colonization in infants post-AZ.

Variable	Adjusted IRR (robust SE)	IRR 95% CI	*P*-value
Treatment arm (BEP plus AZ vs Control)	1.99 (0.41)	(1.33, 2.97)	0.001
Infant LCN-2 3 ng/gm (Q3: 11,696.9-17,731 vs Q1: <7828.3)	0.51 (0.12)	(0.31, 0.81)	0.005
Infant MPO ng/mL (Q3: 4620.8-13,817.5 vs Q1: <785.2)	2.12 (0.57)	(1.25, 3.60)	0.005
Infant MPO (Q2: 785.2-4620.8 vs Q1: <785.2)	2.84 (0.73)	(1.72, 4.69)	<0.001
Maternal sTfR (Q3: 3.7-4.6 mg/L vs Q1: <2.8 mg/L)	0.62 (0.13)	(0.41, 0.96)	0.03
Maternal sTfR (Q4: >4.6 mg/L vs Q1: <2.8 mg/L)	0.53 (0.14)	(0.31, 0.91)	0.02
Colostrum (given to infant within 24 h of birth vs after 24 h of birth)	2.05 (0.39)	(1.41, 2.98)	<0.001
Mode of delivery (vaginal vs C-section)	2.43 (0.54)	(1.58, 3.76)	<0.001

Variables maternal biomarkers (FER, Hb, AGP, CALPR, LIPO, CRP, and MPO), infant biomarkers (FER, Hb, StfR, AGP, CALPR, CRP), place of delivery, and antibiotic prescribed to infant due to illness during the study were initially included in the model but were removed during the backward selection process due to nonsignificance.

The most significant maternal biomarker predictor of infant *B. infantis* colonization was sTfR.

In infants whose mothers had elevated sTfR (>5.4 mg/L, indicating significant anemia), *B. infantis* colonization was reduced by 38% (adjusted RR 0.62; 95% CI: 0.41-0.96) as compared to infants whose mothers had normal sTfR.

Our results show 2.05-fold increase in *B. infantis* colonization in infants who received colostrum within 24 h of birth, as compared to infants who did not (95% CI: (1.41, 2.98). Vaginal delivery was associated with 2.43-fold increase (95% CI: 1.58, 3.76) in odds of *B. infantis* colonization, as compared to C-section delivery (Table 3).

Place of delivery, maternal age, and anthropometric measures were not found to be associated with *B. infantis* colonization of the infant gut.

### Effect of AZ on bacterial count in infants at day 56 postbirth

From the 26 bacteria targets on the TAC card, the mean bacterial count per infant sample was 1.8 ± 1.7. The highest number of bacteria was 7 (3 infants, 2 in arm 1, and 1 in arm 3), and the lowest was 0 (48 infants, 16 in each arm). For every bacteria increase in the mother's stool, the bacterial count in the infant increased by 20% (95% CI: 1.10-1.30). The highest quartile of infant MPO (>13,817 ng/mL) was associated with high bacterial count. The bacterial count for infants with WLZ <−2 (infants with wasting post-AZ) was 1.43 times the bacterial count for infants with WLZ ≥−2 (95% CI: 1.00-2.03). Maternal biomarkers, place and mode of delivery, and concomitant antibiotic use (other than AZ in the treatment arm) were not associated with bacterial count in infants.

### ARGs in infant at day 56 (post-AZ)

The TAC cards were customized to detect 6 antimicrobial genes (ARGs), two from the beta-lactamase *CTX* M family (*ctx_*M*_1_2_9 and ctx_*M*_8_25*), two from the *Escherichia coli/Shigella* fluoroquinolone resistance genes family (*ShEgyrA83L and ShEparC80I*), one *Campylobacter* fluoroquinolone resistance (*Campy23S2075A*) and the macrolide resistance gene mph(A). The prevalence of *CTX-*M type extended-spectrum β-lactamases in this sample of 150 infants was 91.3% (Table S2). The prevalence of mph(A) gene was 68.7% in this cohort. The prevalence of the fluoroquinolone resistance gene with *gyr* mutation was 66.7%. There was no difference between the ARG carriage between infants given AZ and those who were not given AZ.

## Discussion

This study provides epidemiological evidence for the role of AZ in modulating infant infection, inflammation, and growth. Our findings demonstrate that AZ alters the infant microbiome, as evidenced by a 2-fold increase in *B. infantis* colonization, and a reduction in bacterial enteropathogen burden. Additionally, infants who received AZ showed reduced levels of inflammatory biomarkers, such as MPO. Elevated LCN-2 levels (a marker of enhanced inflammation [10]) were associated with a 49% reduction in *B. infantis* colonization (Table 3). These findings underscore the complex interplay between microbiota, inflammation, and antibiotic treatment in early life.

Our study found that a single prophylactic dose of AZ significantly increased *B. infantis* colonization and reduced bacterial enteropathogen burden. This dual effect contrasts with prior studies, such as Wei et al. [11] who reported reduced microbiome diversity and *Bifidobacterium spp*. levels following a 3-day AZ course in Danish children 12-36 months old. However, the specific species affected were not identified. The discrepancies may stem from differences in dosage, timing, and age of administration. Similarly, long-term effects of biannual AZ treatment in Niger [12,13] showed reductions in microbiome richness and diversity, particularly of pathogenic species like *Campylobacter spp*. By contrast, our findings suggest that a single dose of AZ may have a more nuanced impact, increasing *B. infantis* colonization while simultaneously reducing pathogenic bacterial load. Within the AZ group, bacterial counts significantly decreased postadministration compared to pretreatment levels (Table 4), consistent with Parker et al.’s [14] findings in Indian infants, where AZ reduced enteropathogen load after prophylactic AZ. These results support the efficacy of AZ in reducing pathogenic bacterial burden, potentially disrupting the cycle of inflammation-driven pathogen proliferation [15].

**Table 4:** Mean number of bacterial pathogens detected in infant stool by treatment arm pre-AZ and post-AZ.

Study arm	Bacterial pathogen count pre-AZ (mean ± SD)	Bacterial pathogen count post-AZ (mean ± SD)	*P*-value (difference in mean pathogen count pre and post-AZ)^a^
Control	1.84 ± 1.89	1.92 ± 1.94	0.7
BEP alone	1.66 ± 1.56	1.76 ± 1.56	0.8
BEP plus AZ	2.34 ± 2.03	1.82 ± 1.67	0.01
Total		1.83 ± 1.72	

One-way ANOVA was used to compare mean pathogen count by treatment arm. Pre-AZ *P*-value = 0.2 and post-AZ *P*-value = 0.9. Bartlett's equal-variances test showed variances to be equal (*P* = 0.3).

a Paired *t*-test was used to compare the mean number of bacterial pathogens by treatment arm, using a one-sided test of hypothesis (mean pathogen count post-AZ < mean pathogen count pre-AZ).

*In vivo* studies suggest AZ influences human innate immune pathways by reducing Neutrophil Extracellular Traps (NETs) formation [16], MPO, a key enzyme in NET formation, generates reactive oxygen species (ROS) and drives oxidative stress and inflammation [17]. AZ reduces NET release by modulating neutrophil activation, decreasing ROS production, and downregulating pro-inflammatory cytokines like IL-1β and TNF-α. Consistent with these mechanisms, we observed significant reductions in MPO levels post-AZ administration (Figure S1), which likely contributed to mitigating inflammation.

Elevated MPO levels were associated with higher bacterial enteropathogen counts (Table 5), suggesting that persistent inflammation may foster pathogen overgrowth while compromising beneficial microbes like *B. infantis*.

**Table 5:** Factors associated with bacterial count in infants post-AZ.

Variable	Adjusted IRR (robust SE)	IRR 95% CI	*P*-value
Maternal bacterial count	1.20 (0.05)	(1.10, 1.30)	<0.001
Bacterial count pre-AZ	1.36 (0.06)	(1.25, 1.48)	<0.001
Infant MPO (Q4 vs Q1)	1.50 (0.34)	(1.00, 2.34)	0.05
Wasting WLZ (<−2)	1.43 (0.26)	(1.00, 2.03)	0.05

Variables treatment arm, maternal biomarkers (FER, Hb, StfR, AGP, CALPR, LIPO, CRP and MPO), infant biomarkers (FER, Hb, StfR, AGP, CALPR, LIPO, CRP), place and mode of delivery, *Bifidobacterium infantis* colonization and antibiotic prescribed to infant due to illness during the study were initially included in the model but were removed during the backward selection process due to nonsignificance.

In a recent analysis of the MAL-ED study, McCormick et al. [18] demonstrated that breast milk intake was associated with an increase in MPO levels. This rise can be attributed to the known probiotic effect of *B. infantis* in triggering cytokine release and pro-inflammatory responses [19]. This key function of *Bifidobacterium* strains helps to instruct and condition the infant's immune system, thus maintaining health benefits throughout the years [20]. During normal immune function, the body's inflammatory markers, like MPO, rise in moderation. When there is a shift from normality (in cases of persistent infection, inflammation, or malnutrition), the body's inflammatory markers can rise significantly. In our study, *B. infantis* colonization was associated with moderate levels of MPO, but not with the highest quartile of MPO, reflecting the delicate balance between microbial colonization and inflammation.

Correlations between infant MPO, LCN-2, and CALPR reinforce their utility as noninvasive biomarkers of intestinal inflammation, such as in EED [21,22]. Among infant biomarkers, there was a highly significant (*P* < 0.001) positive correlation between infant CRP and AGP, MPO, and LCN-2, and finally, MPO and CALPR (Table S3). The correlation between infant MPO and LCN-2 (Spearman's rho 0.43) and MPO and CALPR (Spearman's rho 0.30, *P* < 0.001) corroborates previous studies [23]. As expected, inverse correlations were noted between FER and sTfR, as well as for Hb and sTfR (*P* < 0.001).

Infant CALPR was weakly associated with maternal CALPR, LCN-2, and sTfR (Spearman's rho 0.2; *P* < 0.05) (Table S4). CALPR and LCN-2 are well-established markers of intestinal inflammation and microbial dysbiosis. Elevated levels of these markers reflect shifts in the gut microbiota and localized inflammatory responses. Their measurement in both maternal and infant stool provides an opportunity to study shared microbiome signatures, likely influenced by vertical transmission pathways such as delivery mode, breastfeeding, and close maternal-infant contact during early life. Understanding these associations is crucial, as they offer insights into how maternal microbiome characteristics and inflammatory states may shape the infant's gut microbiome and impact early health outcomes.

Our study identified a significant association between wasting (WLZ <−2) and higher bacterial enteropathogen counts post-AZ treatment. This is consistent with prior research linking gut microbiome dysbiosis to growth faltering in childhood [24]. Moreover, this analysis was adjusted for concomitant antibiotic use, maternal biomarkers, place, and mode of delivery. Elevated maternal soluble transferrin receptor (sTfR) levels (>3.7 mg/L), indicative of iron deficiency, were associated with reduced *B. infantis* colonization in infants (Table 3). Vertical transmission of bifidobacteria from the mother (vagina, GI tract, or breast milk) has been demonstrated [25]. *B. infantis* predominantly thrives in breastfed infants due to its specific ability to breakdown human milk oligosaccharides (HMO) [26]. Maternal iron deficiency could indicate poor maternal health and nutrition and compromised quantity or quality of breast milk. A reduction in HMOs would likely reflect a reduced colonization of *B. infantis* in infants. Furthermore, our analysis revealed a strong association between maternal and infant bacterial counts, even at 2 months postpartum, regardless of delivery mode, place of birth, or exclusive breastfeeding practices (Table 5). This underscores the role of both vertical and environmental transmission in shaping the infant gut microbiota. Future Mumta sub-studies aim to assess the effect of maternal food intake and BEP supplements on metabolomic profiles (e.g., breast milk nutrient composition).

In our study of 150 infants, we found no difference between AMR gene carriage by treatment arm (Table S2). An important finding was the high prevalence, 60%, of antimicrobial resistance (AMR) genes, particularly mph(A), which encodes a macrolide 2′-phosphotransferase enzyme conferring resistance to AZ [27]. In mph(A) gene carriage between the groups suggests that resistance may result from horizontal gene transfer (HGT) rather than direct antibiotic exposure [28]. While HGT is a plausible explanation, vertical transmission, and environmental factors may also play significant roles. Genomic studies are needed to elucidate the mechanisms underlying AMR gene dissemination.

There are several notable implications of this study. The focus on understanding the role of AZ in *B. infantis* colonization in Pakistani infants is both timely and judicious, given mounting evidence from around the world on numerous beneficial health outcomes conferred through early colonization by this subspecies. In the Mumta primary analysis, we found that postnatal maternal BEP supplementation and infant AZ administration could modestly improve infant growth outcomes at 6 months [29]. This sub-study adds a piece to the puzzle of AZ-related microbiome perturbations and growth. We report MPO levels can signal important shifts in bacterial populations. Further, we show no difference in AMR carriage, of particular interest the mph(A) gene, by treatment arm. Our study contributes important data for the WHO guidelines for the prophylactic use of AZ for the prevention of childhood infections and mortality.

There are some important limitations of this analysis. Our analysis on bacterial count was not stratified by enteropathogen family. We cannot tell which bacteria were affected by a single dose of AZ. Of the 150 mother-infant pairs randomly selected for biomarker and enteropathogen detection, we did not exclude anyone based on existing infections, illnesses, or current use of antibiotics. This information was, however, carefully recorded in the data collection forms, and adjusted for in the analysis.

*In vitro* and *in vivo* studies are needed to elucidate the mechanisms underlying the effects observed in this study and to determine whether they are sustained beyond the 2-week post-treatment period. RCTs during pregnancy may be necessary to confirm these findings and assess their impact on critical outcomes, such as stillbirth and neonatal mortality. Additionally, genetic epidemiology studies should focus on mapping antimicrobial resistance, particularly related to AZ.

## Declarations of competing interest

The authors declare that they have no competing interests. The authors report no conflict of interest.

## Appendix Supplementary materials

Image, application 1

Image, application 2

Image, application 3

Image, application 4

Image, application 5
