# Supplementary material for: Effect of one prophylactic dose of azithromycin on Bifidobacterium infantis colonization in infants from the Mumta trial
Source: Int J Infect Dis. 2025 Apr;153:None. doi: 10.1016/j.ijid.2025.107794 (PMC11910343; doi:10.1016/j.ijid.2025.107794)
Supplement: Supplementary file 1 [file mmc1.docx]

**Pasha et al. (2024). Effect of one prophylactic dose of Azithromycin on *Bifidobacteria infantis* colonization in infants from the Mumta Trial**

Supplementary Figure S1:

Supplementary Figure 1: Boxplot of Infant MPO levels (ng/mL) of stool at two timepoints: pre-AZ (day 42) and post-AZ (day 56). Wilcoxon signed-rank test was used to compare paired data. Differences (Sign test) within same arm at different time point: Difference (p-value): Control (T2) – Control (T1) = 746.5 (0.68); BEP only (T2) – BEP only (T1) = 2411.25 (0.07); BEP plus Azithromycin (T2) – BEP plus Azithromycin (T1): -6283.25 (0.03).
