# Supplementary material for: Effect of one prophylactic dose of azithromycin on Bifidobacterium infantis colonization in infants from the Mumta trial
Source: Int J Infect Dis. 2025 Apr;153:None. doi: 10.1016/j.ijid.2025.107794 (PMC11910343; doi:10.1016/j.ijid.2025.107794)
Supplement: Supplementary file 2 [file mmc2.docx]

**Pasha et al. (2024). Effect of one prophylactic dose of Azithromycin on *Bifidobacteria infantis* colonization in infants from the Mumta Trial**

Supplementary Table S1: Bacterial targets on TAC for objective 2, with frequency in infants Post AZ, and concordance between mother-infant pairs.

|  | **Bacterial Targets on TAC** | **Frequency in infants post AZ (n=150)** | **Number of concordant pairs*** |
| --- | --- | --- | --- |
|  | Aeromonas species | 1 | 148 |
|  | C. difficile tcdA or tcdB | 9 | 142 |
|  | 23S Wild type of Campylobacter | 10 | 132 |
|  | Campylobacter jejuni and Campylobacter coli | 9 | 137 |
|  | Campylobacter_pan | 12 | 127 |
|  | Enteraggregative E.coli (aaic) | 14 | 114 |
|  | Enteraggregative E.coli (aata) | 52 | 107 |
|  | Typical Enteropathogenic E.coli (bfpa) | 5 | 139 |
|  | Atypical Enteropathogenic E.coli (eae) | 11 | 89 |
|  | Enterotoxigenic E.coli (LT, or STh, or STp) | 8 | 127 |
|  | Enterotoxigenic E.coli (STh or STp) | 2 | 141 |
|  | Enterotoxigenic E.coli (LT) | 1 | 145 |
|  | Helicobacter pylori | 0 | 150 |
|  | Mycobacterium tuberculosis | 0 | 150 |
|  | Plesiomonas shigelloides | 1 | 145 |
|  | Salmonella enterica | 0 | 149 |
|  | E.coli/Shigella (gyrA wildtype) | 70 | 107 |
|  | E.coli/Shigella (parC wildtype) | 68 | 107 |
|  | Shigella clade 1 | 1 | 143 |
|  | Shigella species and Enteroinvasive E. coli | 0 | 129 |
|  | Shigella flexneri 6 | 1 | 145 |
|  | Shigella flexneri serotypes excluding serotype 6 | 0 | 148 |
|  | Shigella sonnei | 0 | 146 |
|  | Shigatoxigenic serotypes of E. coli (STEC stx1) | 0 | 147 |
|  | Shigatoxigenic serotypes of E. coli (STEC stx2) | 0 | 150 |
|  | Vibrio cholerae | 0 | 150 |

*Both mother and infant are positive or negative for pathogen
