# Supplementary material for: Effect of one prophylactic dose of azithromycin on Bifidobacterium infantis colonization in infants from the Mumta trial
Source: Int J Infect Dis. 2025 Apr;153:None. doi: 10.1016/j.ijid.2025.107794 (PMC11910343; doi:10.1016/j.ijid.2025.107794)
Supplement: Supplementary file 3 [file mmc3.docx]

**Pasha et al. (2024). Effect of one prophylactic dose of Azithromycin on *Bifidobacteria infantis* colonization in infants from the Mumta Trial**

**Supplementary Table S2:** Antimicrobial resistance genes detected through customized TAC in infant stool by treatment arm Post AZ

| **Antimicrobial resistance gene**  **n (%)** | **N=150** | | **LC (n=50)** | | **LC & BEP (n=50)** | | **LC & BEP/ AZ (n=50)** | | ***P* value** |
| --- | --- | --- | --- | --- | --- | --- | --- | --- | --- |
| ctx_M_1_2_9 | 137 | 91.3% | 45 | 90.0% | 46 | 92.0% | 46 | 92.0% | 0.9 |
| ctx_M_8_25 | 20 | 13.3% | 5 | 10.0% | 9 | 18.0% | 6 | 12.0% | 0.4 |
| Campy23S2075A | 12 | 8.0% | 4 | 8.0% | 5 | 10.0% | 3 | 6.0% | 0.9 |
| Mph(A) | 103 | 68.7% | 31 | 62% | 33 | 66% | 39 | 78% | 0.2 |
| ShEgyrA83L | 100 | 66.7% | 30 | 60.0% | 36 | 72.0% | 34 | 68.0% | 0.4 |
| ShEparC80I | 53 | 35.3% | 16 | 32.0% | 17 | 34.0% | 20 | 40.0% | 0.6 |
|  |  |  |  |  |  |  |  |  |  |

Pearson’s Chi2 test or Fischer’s exact test was used to compare frequencies of antimicrobial resistance genes (ARGs) by treatment arm.
