# Supplementary material for: Effect of one prophylactic dose of azithromycin on Bifidobacterium infantis colonization in infants from the Mumta trial
Source: Int J Infect Dis. 2025 Apr;153:None. doi: 10.1016/j.ijid.2025.107794 (PMC11910343; doi:10.1016/j.ijid.2025.107794)
Supplement: Supplementary file 4 [file mmc4.docx]

**Pasha et al. (2024). Effect of one prophylactic dose of Azithromycin on *Bifidobacteria infantis* colonization in infants from the Mumta Trial**

Supplementary Table S3: Spearman’s correlation matrix for relationship between infant biomarkers.

| **Infant** | **CALPR** | **LCN-2** | **MPO** | **Hgb** | **FER** | **sTfR** | **CRP** | **AGP** |
| --- | --- | --- | --- | --- | --- | --- | --- | --- |
| **CALPR** | 1.00 |  |  |  |  |  |  |  |
| **LCN-2** | 0.26* | 1.00 |  |  |  |  |  |  |
| **MPO** | 0.30** | 0.43** | 1.00 |  |  |  |  |  |
| **Hgb** | 0.11 | 0.03 | 0.04 | 1.00 |  |  |  |  |
| **FER** | -0.02 | -0.03 | -0.12 | 0.08 | 1.00 |  |  |  |
| **sTfR** | -0.07 | 0.00 | 0.04 | -0.27* | -0.31** | 1.00 |  |  |
| **CRP** | 0.09 | 0.19 | 0.15 | -0.09 | 0.08 | 0.08 | 1.00 |  |
| **AGP** | 0.09 | 0.08 | 0.08 | -0.01 | 0.17 | 0.17 | 0.67** | 1.00 |

* *P* value<0.05; ***P* value<0.001. Spearman’s correlation was used to assess the strength of association between maternal and infant biomarkers. Negative linear relationship is denoted with (-) sign. Spearman’s rho 0.4-0.69 is taken as strong relationship between the biomarkers.
