# Supplementary material for: Effect of one prophylactic dose of azithromycin on Bifidobacterium infantis colonization in infants from the Mumta trial
Source: Int J Infect Dis. 2025 Apr;153:None. doi: 10.1016/j.ijid.2025.107794 (PMC11910343; doi:10.1016/j.ijid.2025.107794)
Supplement: Supplementary file 5 [file mmc5.docx]

**Pasha et al. (2024). Effect of one prophylactic dose of Azithromycin on *Bifidobacteria infantis* colonization in infants from the Mumta Trial**

Supplementary Table S4: Spearman’s correlation coefficient matrix for biomarkers of mother and infant (Post-AZ).

| **Infant↓** | **Maternal→** | **CALPR** | **LCN-2** | **MPO** | **Hgb** | **FER** | **sTfR** | **CRP** | **AGP** |
| --- | --- | --- | --- | --- | --- | --- | --- | --- | --- |
| **CALPR** | | 0.2* | 0.2* | -0.02 | 0.008 | -0.08 | 0.2* | 0.01 | 0.1 |
| **LCN-2** | | 0.06 | 0.02 | 0.05 | 0.03 | -0.07 | -0.13 | -0.10 | -0.2* |
| **MPO** | | 0.04 | -0.03 | -0.20 | -0.03 | -0.02 | 0.14 | -0.04 | -0.01 |
| **Hgb** | | -0.01 | 0.002 | -0.07 | -0.008 | -0.1 | 0.1 | -0.2 | -0.1 |
| **FER** | | 0.08 | 0.1 | 0.20 | -0.04 | -0.04 | 0.13 | 0.01 | 0.1 |
| **sTfR** | | 0.005 | -0.02 | 0.07 | 0.01 | 0.07 | -0.03 | 0.01 | 0.1 |
| **CRP** | | -0.07 | 0.08 | -0.01 | -0.01 | 0.04 | -0.06 | 0.10 | 0.02 |
| **AGP** | | -0.04 | 0.1 | 0.05 | -0.04 | 0.03 | -0.01 | 0.01 | 0.1 |

**P* value<0.05. Spearman’s correlation was used to assess the strength of association between maternal and infant biomarkers. Negative linear relationship is denoted with (-) sign. Spearman’s rho of 0.2-0.39 is taken as weak-moderate relationship.
